# Enhancing Stability and Performance of Conductive Bridge Random Access Memory: Use of a Copper-Doped ZnO Nanorod-Embedded Switching Layer

**DOI:** 10.1021/acsami.4c16223

**Published:** 2025-02-12

**Authors:** Po-Tsun Liu, Yu-Chuan Chiu, Chih-Chieh Hsu, Kai-Jhih Gan, Dun-Bao Ruan, Sheng-Jie Su, Shu-Wei Chang

**Affiliations:** † Department of Photonics and Institute of Electro-Optical Engineering, National Yang Ming Chiao Tung University, Hsinchu 300093, Taiwan; ‡ Institute of Electronics, National Yang Ming Chiao Tung University, Hsinchu 300093, Taiwan

**Keywords:** ZnO, nanorod, hydrothermal method, resistive random access memory, conductive bridge random access memory, ECM type

## Abstract

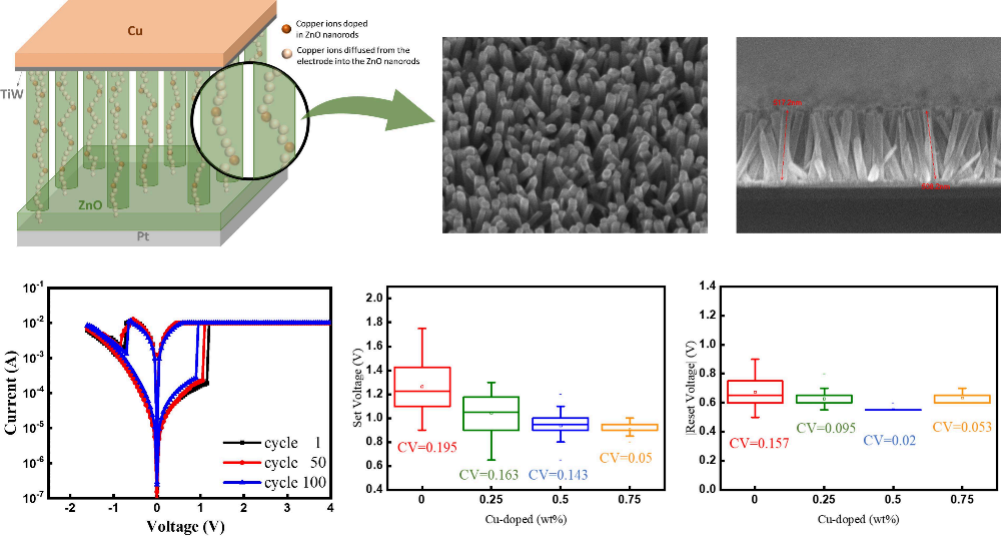

Computing-in-memory (CIM) technology for edge computing systems demands memory devices that are not only fast but also capable of being easily integrated into stackable manufacturing processes. To address these requirements, conductive bridge random access memory (CBRAM) devices, which are compatible with copper-wiring processes, have emerged as promising candidates. However, the practical implementation of CBRAM is hindered by challenges associated with physical defects in its operating mechanism, leading to issues such as non-uniform switching voltages and high energy consumption in high-density storage applications. This study introduces a layer of copper-doped zinc oxide (ZnO) nanorods as the switching medium in the CBRAM device. The nanostructures play a critical role in regulating copper ion diffusion, thereby facilitating the formation of uniform conductive filaments. The influence of ZnO nanorods on the copper ion diffusion behavior and filament morphology was analyzed through characteristic current fitting of CBRAM devices. Experimental results demonstrate that ZnO nanorod-embedded CBRAM exhibits significantly enhanced memory performance, characterized by superior switching uniformity compared to devices lacking ZnO nanorods. Furthermore, CBRAM devices incorporating copper-doped ZnO nanorods demonstrate even greater memory performance, achieving exceptional uniformity. This approach not only reduces operating voltage and energy consumption but also improves switching uniformity and enhances the overall stability of CBRAM devices, making them more viable for advanced CIM applications.

## 1 Introduction

The establishment of extensive cloud computing data centers and the enhancement of edge device performance are imperative for the operation of complex artificial intelligence (AI) functionalities. To store and process vast amounts of data under the premise of low energy consumption, data centers urgently require novel non-volatile memory (NVM) storage technologies that offer higher density and lower cost. On the other hand, edge devices encounter two key challenges: “high latency” and “power consumption of multiply accumulate (MAC) operations”. The introduction of processing-in-sensor (PIS), compute-in-memory (CIM),^1−3^ and convolutional neural network (CNN)^4,5^ processor technologies is widely regarded as the optimal solution to address these issues. Among these technologies, CIM enables direct execution of AI computations on memory components, thereby reducing the speed limitations and energy consumption caused by data transfer between memory and processors in traditional von Neumann architecture. Resistive random access memory (RRAM),^6^ due to its simple structure, scalability, low power consumption, rapid operation, and long lifespan, can meet the modern technological demand for high-density storage. Moreover, RRAM’s simple fabrication process facilitates its integration with other components, making it suitable for forming multifunctional circuits.^7^ In recent years, RRAM has been frequently employed in CIM technology to enhance the performance of edge devices. Given its operation mode resembling that of neural synapses, RRAM can be utilized as a biomimetic component in neuromorphic circuits,^8^ and it finds extensive applications in artificial intelligence chips and biomedical fields.^9−11^

RRAM operates by applying a bias voltage to form conductive filaments in the switching layer. Once the filaments are formed, the device switches to a low-resistance state with high current flow.^12^ Applying a reverse voltage at this point severs the conductive filaments, returning the device to its initial high-resistance state with low current flow. Dependent upon the dominant mechanism governing filament formation during operation, RRAM can be categorized into oxide-based random access memory (OxRAM), where filament formation is primarily driven by oxygen vacancies, and conductive bridge random access memory (CBRAM), where conductive filaments are formed through the diffusion of electrode metal ions into the switching layer. CBRAM typically employs silver or copper as electrode materials.^13,14^ This aligns well with modern electronic technology’s copper wiring techniques, enjoying the advantages of easy fabrication of high-reliability, low-latency, small-size metal interconnects. However, due to the mechanism of utilizing metal ion diffusion to generate conductive filaments in CBRAM operation, uneven filament formation within the switching layer can easily occur due to the stochastic nature of diffusion, resulting not only in unstable operating voltages but also affecting the device’s lifespan. At high-density usage, this can lead to challenges in circuit operation design and impact the lifespan of end products. Some research focuses on optimizing the switching layer to improve the instability of filament formation,^15−17^ while others explore using dual-layer switching layers to enhance the stability of device switching.^17,18^ Additionally, some studies employ nanostructures to confine the formation path of filaments.^12,19−21^ Zinc oxide (ZnO) materials have been extensively utilized in various electronic devices, such as thin-film transistors (TFTs), gas sensors, and ultraviolet (UV) sensors, owing to their wide bandgap, high optical transparency in the visible spectrum, and excellent carrier mobility. In the field of RRAM, ZnO thin films and nanorods are frequently employed as switching layers due to their facile synthesis, cost-effectiveness, and advantageous distribution of oxygen vacancies.^22^ Previous studies on ZnO nanorod-based RRAM devices have primarily focused on enhancing their performance by modulating oxygen vacancies, a key factor influencing resistive switching behavior. To achieve this, researchers have explored strategies such as doping ZnO nanorods with various elements to optimize their electrical and structural properties.^19−21^ Recently, as CBRAM has gained increasing attention, ZnO nanorods have also been investigated as potential switching layers in CBRAM architectures, further expanding their applicability in advanced memory technologies.^12,23,24^

In previous studies, we observed significant enhancement in RRAM device stability by utilizing zinc oxide nanorods to confine filament formation.^19−21^ However, the crystallization, distribution, width, and length of nanorods have a significant impact on CBRAM device characteristics, with thicker nanowires still having the potential to form uneven filaments. To address this issue, in this study, we employed a hydrothermal method to grow high-quality and delicate zinc oxide nanorods,^25^ while incorporating copper ions into the ZnO nanorods during the growth process to ensure uniform distribution. Previous studies solely focused on the use of pure ZnO nanostructures or copper-doped ZnO thin films without exploring the effects of combining of both copper doping techniques and nanorod structures. By combining its physical structure of ZnO nanorods with uniform copper ion distribution, this work can maximize the uniformity of conductive filament formation, thereby enhancing the stability of CBRAM device operation.

## 2 Experimental Section

### 2.1 Device Fabrication

**Figure 1:**
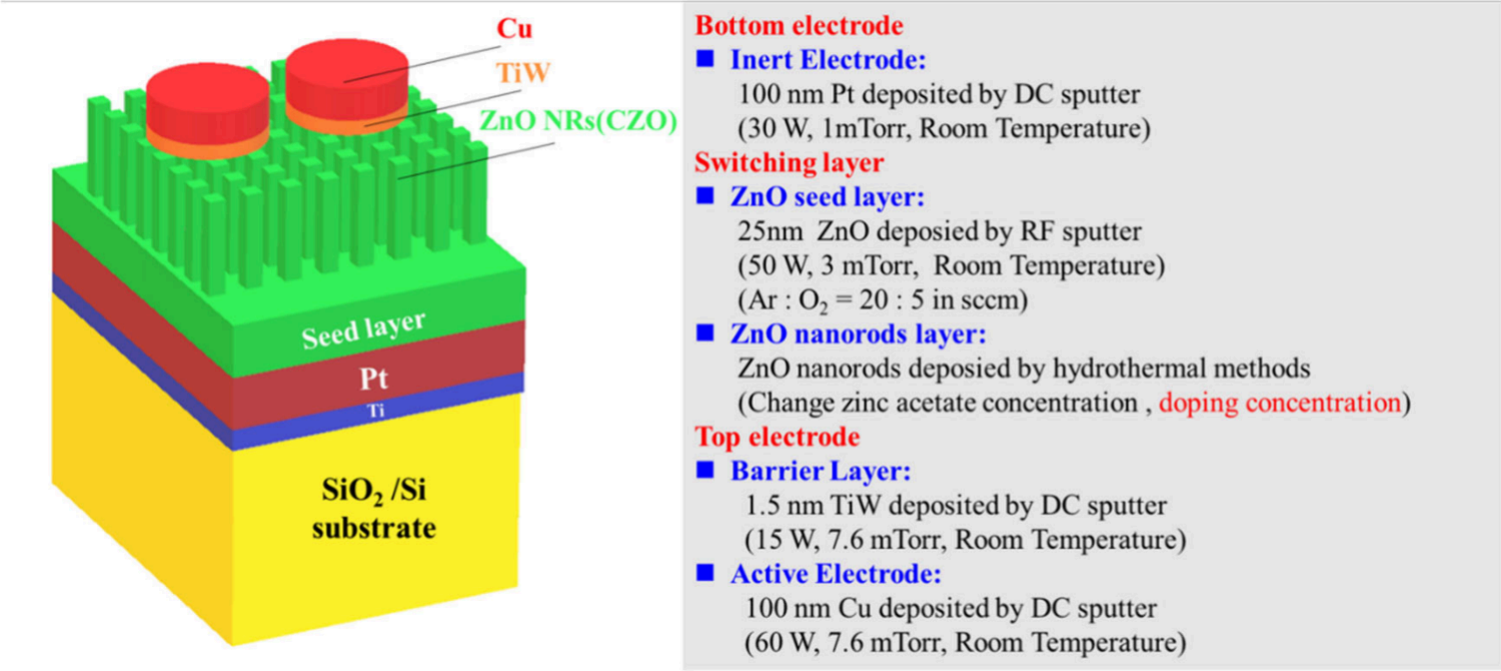
Fabrication of CBRAM device with ZnO nanorods acting as the switching layer. On the left is a schematic diagram of the CBRM device structure with a switching layer of ZnO nanorods. First, a buffer SiO_2_ layer was deposited on the silicon substrate. Then, Ti was deposited as an adhesion layer followed by Pt as the bottom electrode metal. Next, ZnO was deposited as a seed layer via sputtering, and ZnO nanorods were grown using the hydrothermal method. A thin layer of TiW was deposited as a diffusion barrier layer before the copper electrode metal was deposited as the top electrode.

To address variations in copper ion concentrations within resistance switching layers, this study focuses on the fabrication of copper-doped ZnO nanorods using the hydrothermal method. The process begins with the deposition of a 5 nm thick titanium adhesion layer onto a SiO_2_/Si substrate via direct current (DC) sputtering. Subsequently, a 100 nm thick platinum layer is sputtered onto the titanium layer to serve as the bottom electrode. Prior to the hydrothermal growth of ZnO nanorods, a 25 nm thick ZnO seed layer is deposited onto the bottom electrode using radio frequency (RF) magnetron sputtering. The sputtering process is conducted under fixed parameters, including an RF power of 50 W and a process pressure of 3 mTorr, with an Ar/O_2_ gas flow ratio of 20:5 standard cubic centimeters per minute (sccm). For the hydrothermal synthesis process,^26^ we utilized zinc acetate dihydrate [Zn(OOCCH_3_)_2_·2H_2_O, purity of 99.9%] and hexamethylenetetramine (HMTA, C_6_H_12_N_4_, ACS grade, purity of 99.9%).^26,27^ Additionally, copper(II) acetate [ACS grade, copper(II) acetate monohydrate, 98–102%] was employed as a dopant. Aqueous solutions with different concentrations of copper ions (0.02 M) were prepared at room temperature. Subsequently, the substrates were immersed in the solutions within a beaker using a sample holder, and the growth temperature was maintained at 90 °C for 30 min. Finally, a 1.5 nm thick TiW barrier layer and a 100 nm thick copper top electrode were sequentially deposited on the switching layers.^28^ The active copper electrode, 100 μm in diameter, was cylinder-shaped and patterned using a shadow mask. The completed copper-doped zinc oxide nanorod-based CBRAM devices are illustrated in Figure 1.

### 2.2 Hydrothermal Method (Solvent-Thermal Method)

The detailed fabrication steps of the hydrothermal process are as follows: Initially, prepare the process solution in a beaker and mix it thoroughly using a stirrer. In the hydrothermal process solution for ZnO nanorods, zinc ions are sourced from zinc acetate dihydrate [Zn(OOCCH_3_)_2_·2H_2_O], and hydroxide ions come from hexamethylenetetramine (C_6_H_12_N_4_). The zinc ions and hydroxide ions are dissolved in deionized water in a 1:1 ratio. At this stage, an appropriate amount of copper acetate is added to the beaker based on the impurity concentration of copper ions. Subsequently, the substrate with the deposited seed layer undergoes ultrasonic vibration cleaning and is dried using a nitrogen gun. The substrate is then placed on a microscope slide and immersed in the process solution at a 70° angle^29^ with the container sealed. The container is placed on a heating platform to initiate the growth of the nanorods, with the process temperature set at 90 °C and maintained within a ±2 °C range. After the reaction, the substrate is removed, washed with deionized water to remove impurities, and allowed to air dry naturally. In this study, to investigate the fabrication of zinc oxide nanorods and their effects on devices, experiments were conducted with four different concentrations of zinc acetate ranging from 0.005 to 0.08 M. Growth conditions for undoped nanorods were also tested at three different time intervals: 30, 60, and 90 min. For copper ion doping, eight different concentrations ranging from undoped to 2 wt % were studied. The reaction process in this experiment utilizing the water bath method is illustrated by the following equations:
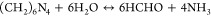
1
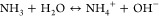
2
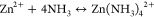
3
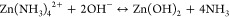
4
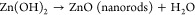
5

### 2.3 Characterization

In this work, all zinc oxide nanorod CBRAM devices were characterized using a semiconductor parameter analyzer (Keithley 4200). X-ray photoelectron spectroscopy (XPS) analysis was conducted using a Thermo Fisher Scientific Theta Probe instrument. X-ray diffraction (XRD) measurements were performed using the Bede D1 instrument. Field emission scanning electron microscopy (FESEM) imaging was carried out using the JEOL JSM-6700F.

## 3 Results and Discussion

### 3.1 Investigation of Preparation Conditions and Properties of ZnO Nanorods

**Figure 2:**
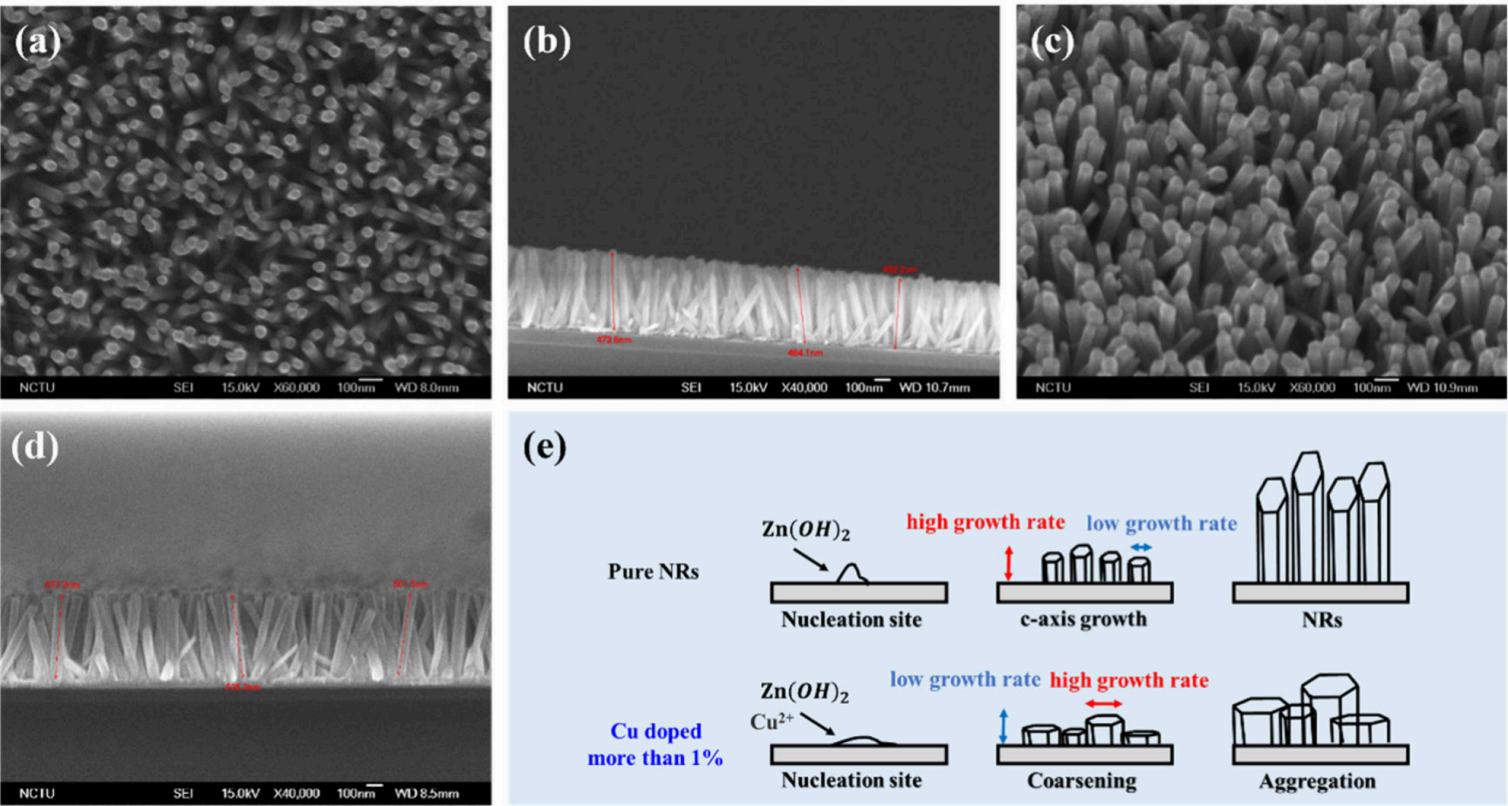
SEM images of ZnO nanorods under optimal conditions and the impact of copper doping on nanorod morphology. (a) Top-view SEM image of ZnO nanorods synthesized with a molar concentration of 0.02 M zinc acetate without copper doping, showing a nanorod diameter of approximately 25 nm. (b) Cross-sectional SEM image of ZnO nanorods synthesized with a molar concentration of 0.02 M zinc acetate without copper doping, showing a nanorod height of approximately 450 nm. (c) Top-view SEM image of ZnO nanorods synthesized with a molar concentration of 0.02 M zinc acetate doped with 0.75% copper, showing an increased nanorod diameter of approximately 50 nm. (d) Cross-sectional SEM image of ZnO nanorods synthesized with a molar concentration of 0.02 M zinc acetate doped with 0.75% copper, showing an increased nanorod height of approximately 500 nm. (e) These results suggest that copper ion doping reduces the vertical growth rate of the nanorods while enhancing their lateral growth rate.

To investigate the influence of different concentrations of zinc acetate on the growth of ZnO nanorods, this study employed four different molar concentrations of zinc acetate, namely, 0.005, 0.02, 0.05, and 0.08 M, to grow zinc oxide nanorods. The top-view scanning electron microscopy (SEM) images of the nanorod morphology are displayed in Figure S1 of the Supporting Information. From the SEM images, it was observed that under lower concentrations of zinc acetate, the zinc oxide nanorods exhibited poorer vertical alignment and a looser structure. With the increase in zinc acetate concentration, the vertical alignment of the nanorods improved, and the density became denser. However, excessively high concentrations of zinc acetate led to the nanorods becoming overly stout, and even overlapping with each other, resulting in a morphology more akin to a film rather than individual nanorods. The observed phenomenon can be explained by the role of zinc acetate concentration in controlling the initial aggregation of ZnO nanoparticles. At low zinc acetate concentrations, the aggregation of ZnO nanoparticles during the initial nucleation phase is minimal.^30,31^ This leads to the formation of small, isolated nucleation centers, which in turn result in nanorods with smaller radii. These slender nanorods lack sufficient structural support during growth, making them prone to collapse and compromising the uniformity of their vertical alignment. In contrast, higher concentrations of zinc acetate promote faster aggregation of ZnO nanoparticles, resulting in the formation of larger nucleation centers. This facilitates the growth of nanorods with increased radii, which provides enhanced structural stability as their vertical length increases. Consequently, ZnO nanorods grown under these conditions exhibit improved vertical alignment and greater uniformity. In order to utilize the nanorod structure to regulate the diffusion of copper ions, uniformity in filament formation needed to be increased. Based on the results in Figure S1 of the Supporting Information, we evaluated ZnO nanorods grown at 0.02 M zinc acetate concentration as the optimal condition because of their excellent vertical alignment and appropriate density. Their top-view and cross-sectional SEM images are shown in panels a and b of Figure 2, respectively. Furthermore, we investigated the influence of different growth times on nanorod growth. Figure S2 of the Supporting Information illustrates the nanorod growth for 30, 60, and 90 min at a zinc acetate concentration of 0.02M. Analysis of the heights measured from cross-sectional images revealed that the height of the nanorods exhibited a nonlinear increase with extended growth time. Simultaneously, top-view observations indicated that the vertical alignment of the nanorods deteriorated as their height increased, accompanied by a tendency for the distribution of nanorods at the top to become increasingly disordered with greater height. To maintain the optimal quality of the nanorods and simultaneously reduce processing time, 30 min was chosen as the standard growth time for the nanorods. In addition, the doping effect by adding copper acetate to the zinc acetate solution was also explored. A total of 7 wt % of copper acetate was used in this work, including 0.1, 0.25, 0.5, 0.75, 1, 1.5, and 2%. The top-view and cross-sectional SEM images illustrating the nanorod growth results under varying copper ion doping concentrations are presented in Figures S3 and S4 of the Supporting Information, respectively. From Figure S3 of the Supporting Information, it can be seen that at copper ion doping concentrations below 1%, the SEM top-view images show no significant changes in the morphology of the ZnO nanorods. The preferable condition with excellent vertical alignment and appropriate density was found to be a doping concentration of 0.75% copper ions, with SEM top-view and cross-sectional images presented in panels c and d of Figure 2, respectively. However, when the doping concentration exceeded 1%, an increase in the diameter of the nanorods was observed, leading to overlapping and densification. These phenomena were validated by the SEM cross-sectional images in Figure S4 of the Supporting Information. Initially, Figure S4 of the Supporting Information shows that with a fixed growth time of 30 min, an increase in copper ion doping concentration results in a decrease in the length of the nanorods. This indicates a reduced growth rate as the copper ion doping concentration increases. At high concentrations, the SEM cross-sectional images reveal that the nanorod morphology is almost absent, instead showing overlapping structures that resemble a film rather than distinct nanostructures. The observed effects of Cu doping on the growth of ZnO nanorods can be attributed to several interrelated factors.^32−34^ According to the literature, the incorporation of Cu into ZnO does not significantly alter the crystal structure. However, the presence of Cu promotes the formation of nanorods with a hexagonal rod-like shape. This shape preference arises from the influence of Cu ions on the surface energy and growth dynamics of ZnO crystals. As the concentration of Cu^2+^ ions in the solution increases, the growth of ZnO nanorods is inhibited. This is reflected in a reduction in the length of the nanorods and an increase in their width, leading to a thicker morphology. The inhibitory effect is likely due to Cu ions partially occupying active growth sites on the ZnO crystal surface, thereby slowing down the growth along the *c*-axis. Simultaneously, the reactions of copper acetate in water, as shown in eqs 6 and 7, increase the concentration of OH^–^ ions in the solution.
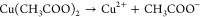
6
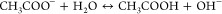
7The increased OH^–^ concentration accelerates the nucleation and initial growth of ZnO nanorods, contributing to their thicker morphology. When the Cu doping concentration is below 1%, the inhibitory effects of Cu ions on axial growth and the acceleration of growth due to increased OH^–^ are nearly balanced. As a result, the nanorods adopt a thicker and more hexagonal shape without significant changes in length. However, when the doping concentration exceeds 1%, the inhibitory effects dominate, leading to a marked reduction in nanorod length and causing them to appear thicker and more film-like in morphology.

To confirm the crystallinity of the zinc oxide nanorods and understand the doping effects of copper ions on the crystallinity, we conducted XRD analysis on samples grown for 30 min under all parameters. The results are presented in Figure S5 of the Supporting Information. By comparison of the peak positions with 2θ values to Joint Committee on Powder Diffraction Standards (JCPDS) card number 36-1451, it was observed that zinc oxide nanorods exhibited diffraction peaks corresponding to the (002), (102), and (103) crystal planes. Among these, the (002) diffraction peak exhibited the highest intensity, indicating that the nanorods are predominantly oriented along this crystal direction. We hypothesize that this phenomenon arises due to the wurtzite structure of ZnO nanorods, which possesses polar (001) facets and nonpolar facets (101) and (100). Crystal facets with higher surface energy exhibit faster growth rates; consequently, the growth rate along the (001) direction exceeds that of the (100) direction. As a result, the (002) diffraction peak becomes the dominant feature in the XRD pattern of the growing ZnO nanorods. The average crystallite size was calculated by correlating the full width at half maximum (fwhm) of the diffraction peaks with their corresponding 2θ positions, using the Debye–Scherrer equation, as shown in eq 8
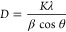
8where *D* represents the average crystalline size, *K* is known as Scherrer’s constant, λ is the X-ray wavelength, β is the line broadening at fwhm, and θ is the Bragg angle. In the experiments, the X-ray wavelength and Bragg angle were 1.54 Å and 34.56°, respectively. Additionally, eq 9(35,36) was used to estimate the residual microstrain
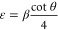
9where ε represents the residual microstrain, β is the line broadening at half the maximum intensity (fwhm), and θ is the Bragg angle. With these parameters as a basis, we conducted our analysis and present the relevant results in Table 1. From Figure S5 of the Supporting Information, it is evident that whether copper ions are doped or not, there are no additional crystal planes observed in the material. This indicates that the doping of copper ions does not lead to the formation of copper oxide or any other compounds derived from copper within the material. From Table 1, it is clear that in samples doped with copper ions, the intensity of the (002) plane decreases compared to pure ZnO nanorods. However, the intensity remains significant up to a doping concentration of 0.75%, indicating a gradual reduction in the (002) diffraction peak intensity with increasing copper ion doping. Furthermore, as shown in Table 1, it is evident that increasing copper doping to a certain proportion (>1%) leads to a noticeable decrease in the intensity of crystal plane diffraction peaks. This finding aligns to some extent with observations from FESEM analysis. Additionally, as shown by the *D* values in Table 1, it is clearly observed that when the copper ion doping concentration is below 2%, the average crystalline size of the ZnO nanorods decreases with increasing doping concentration. This phenomenon can be attributed to the fact that copper ion doping reduces the nucleation and growth rates along the (002) direction. On the basis of the XRD and SEM results, we propose that the lateral growth of ZnO nanorods, as illustrated in Figure 2e, is induced by copper ion doping. Introducing Cu doping agents into the reaction pathway increases the lateral nucleation density, inhibiting growth along the *c*-axis direction. This initially leads to the thickening of nanorods and eventually results in their lateral aggregation. Additionally, it is observed that residual microstrain increases with higher doping concentrations. Typically, larger residual microstrain implies more grain boundaries within the structure, which usually signifies more defects and leads to increased resistivity. In other words, excessive metal doping within zinc oxide results in higher structural resistivity, indicating that more copper ion doping is not necessarily advantageous.

**Table 1:** Information of Fitting the XRD Patterns for the ZnO Nanorods with Copper Doping Concentrations^a^

sample (Cu % doping)	height (a.u.)	fwhm (β) (deg)	2θ (deg)	*D* (nm)	ε (×10^–3^)
0	2022	0.451	34.461	21.38	0.749
0.1	1597	0.454	34.457	21.26	0.753
0.25	1501	0.459	34.458	21.02	0.762
0.5	1614	0.464	34.460	20.79	0.770
0.75	1767	0.469	34.448	20.55	0.779
1	1373	0.475	34.453	20.30	0.789
1.5	737	0.521	34.454	18.53	0.864
2	684	0.565	34.441	17.07	0.939

a Height represents the peak value detected by the diffractometer after diffraction. β is the line broadening at half the maximum intensity, also known as the fwhm. θ is the Bragg angle. *D* is the average crystalline size. ε is the microstrain.

### 3.2 Discussion on Electrical Characteristics of RRAM Device

**Figure 3:**
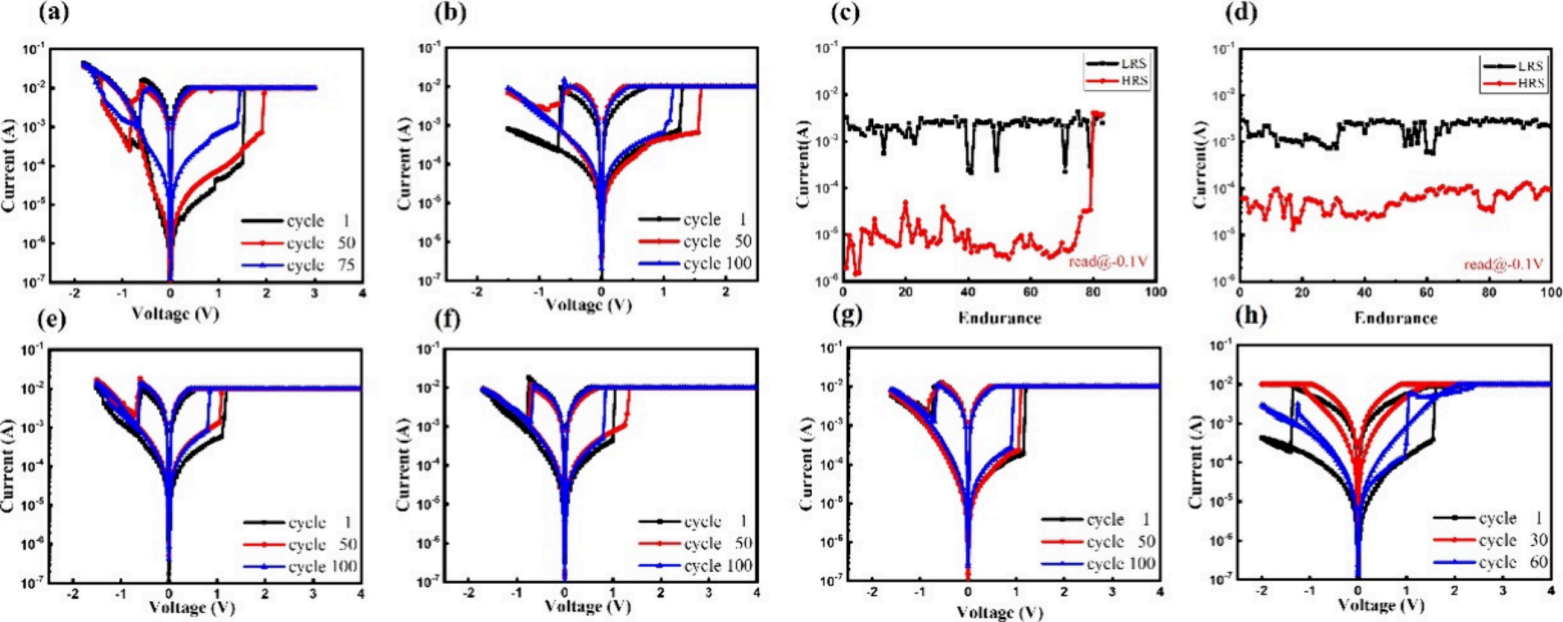
Endurance operation tests of CBRAM devices. (a) Repetitive operation test of ZnO thin film-based CBRAM for 75 cycles. (b) Repetitive operation test of ZnO nanorod-based CBRAM for 100 cycles. (c) Current readings for the high and low resistance states during repetitive operation of ZnO thin film-based CBRAM at −0.1 V. (d) Current readings for the high and low resistance states during repetitive operation of ZnO nanorod-based CBRAM at −0.1 V. (e) Repetitive operation test of ZnO nanorod-based CBRAM with 0.25% copper doping for 100 cycles. (f) Repetitive operation test of ZnO nanorod-based CBRAM with 0.5% copper doping for 100 cycles. (g) Repetitive operation test of ZnO nanorod-based CBRAM with 0.75% copper doping for 100 cycles. (h) Repetitive operation test of ZnO nanorod-based CBRAM with 1% copper doping for 60 cycles.

Figure 3a presents the *I*–*V* curves of CBRAM device with the sole ZnO thin-film acting as a resistive switch layer under repeated electrical operations, while Figure 3b illustrates the ones of CBRAM with undoped ZnO nanorods. The high resistance states (HRS) and low resistance states (LRS) of the two types of CBRAM were measured for each operational cycle and compiled into endurance charts, as shown in panels c and d of Figure 3, respectively. From panels a and b of Figure 3, it is evident that the incorporation of ZnO nanorods enhances the stability of the set/reset voltages and the current values of both HRS and LRS during CBRAM operation, compared to CBRAM devices utilizing only a ZnO thin film as the switching layer. Additionally, as shown in panels c and d of Figure 3, the resistance variation per cycle in the thin-film-based RRAM is significantly larger, with even basic endurance of 100 cycles proving unattainable. However, the incorporation of ZnO nanorod structures significantly reduces variability, indicating their effectiveness in enhancing the uniformity of filament paths. Furthermore, ZnO thin films deposited via sputtering may exhibit lower crystallinity, resulting in higher resistance due to their predominantly amorphous structure. In contrast, ZnO nanorod structures, which typically possess single-crystal characteristics, demonstrate superior conductivity. This explains the increase in the current value of the HRS during CBRAM operation, from 10^–6^–10^–5^ to 10^–5^–10^–4^ A, following the incorporation of nanorod structures into the ZnO film layer. Panels e–h of Figure 3 present the *I*–*V* curves of ZnO nanorod-embedded CBRAM devices prepared with varying copper ion doping concentrations. The endurance charts, depicting the high and low resistance states for each operational cycle, are provided in Figure S6 of the Supporting Information. As shown in panels e–g of Figure 3, the operational voltage of the ZnO nanorod-embedded CBRAM device decreases with increasing copper ion doping. For example, the current value of high-resistance state decreases from 5.83 × 10^–5^ to 2.11 × 10^–5^ A when comparing extreme doping concentrations, such as 0.75% and pure nanorods, which is consistent with the material analysis results. This reduction is attributed to the increased resistance of ZnO nanorods due to copper ion doping. However, Figure 3h shows that doping concentrations exceeding 1% significantly alter the nanorod morphology, leading to shorter filament paths or a more film-like structure, which complicates the writing and erasing processes. Additionally, Figure S6 of the Supporting Information demonstrates a marked deterioration in endurance, with higher doping concentrations, such as 1.5 and 2%, causing the device to fail to maintain conductivity after even 10 cycles.

Oxygen vacancies play a pivotal role in the functioning of RRAM devices, including both OxRAM and CBRAM. To gain deeper insights into the effects of copper doping on ZnO nanorods and to elucidate the reasons for the stability of these devices, we conducted XPS analysis, focusing on oxygen bonding. XPS was also employed to detect the successful incorporation of copper into the sample. However, the minimum detectable limit of copper in the XPS machine is approximately 1 atomic %, precluding the detection of copper signals in samples with lower doping concentrations. Detailed analysis of the XPS spectrum of oxygen O_1s_ involved fitting and deconvoluting the core O_1s_ curves into three Gaussian-distributed peaks around 530 eV binding energies.^37,38^ The peak at 529.8 eV (O_I_) corresponds to oxygen atoms in strong bonds with other atoms, referred to as “oxygen lattice”. The peak at 531.1 eV (O_II_) represents “oxygen vacancies”, indicative of defect states generated by oxygen.^16,39−41^ The peak at 531.8 eV (O_III_), termed “oxygen hydroxide”, corresponds to weak oxygen bonds with adsorbed H_2_O or O_2_ on the bulk surface. Figure S7 of the Supporting Information illustrates the XPS surface analyses of O_1s_, with the fitted raw data results presented in Table 2. Table 2 shows that with increasing copper ion doping concentration, the proportion of oxygen lattice decreases, while the proportion of oxygen vacancies increases. This finding corroborates our previous XRD results, attributing the decrease in oxygen lattice proportion to the disruption of zinc oxide crystallinity by copper doping. Although oxygen vacancies are not the primary component of conductive filaments in CBRAM, their presence facilitates the diffusion of copper ions, forming conductive filaments.^42,43^ However, doping concentrations exceeding 1% lead to severe deterioration of device electrical properties, indicating the need for an appropriate, not excessive, oxygen vacancy content. Additionally, maintaining the independence and vertical alignment of nanorods is crucial to enhancing the uniformity and stability of device operation.

**Table 2:** Fitting Information for XPS Surface Analysis of O 1s for the ZnO Nanorods with Different Copper Doping Concentrations^a^

sample	NR	0.1%	0.25%	0.5%	0.75%	1%	1.5%	2%
O_lattice_ (%)	66.20	63.68	63.17	62.29	61.25	61.52	64.13	64.84
O_vacancy_ (%)	22.50	24.55	25.48	25.95	26.67	26.97	27.08	29.01
O–H (%)	11.30	11.77	11.35	11.76	12.08	11.51	8.79	6.15

a The XPS spectrum of the O 1s core level was de-convoluted into three Gaussian-distributed peaks centered around binding energies of 530 eV. The first peak (O1), located at 529.8 eV, corresponds to oxygen atoms strongly bonded to other elements, referred to as the “oxygen lattice”. The second peak (O_2_), observed at 531.1 eV, represents oxygen vacancies and is associated with defect states induced by oxygen. The third peak (O_3_), appearing at 531.8 eV, is attributed to the “oxygen hydroxide” bond and reflects weak bonding of oxygen with adsorbed H_2_O or O_2_ on the surface.

**Figure 4:**
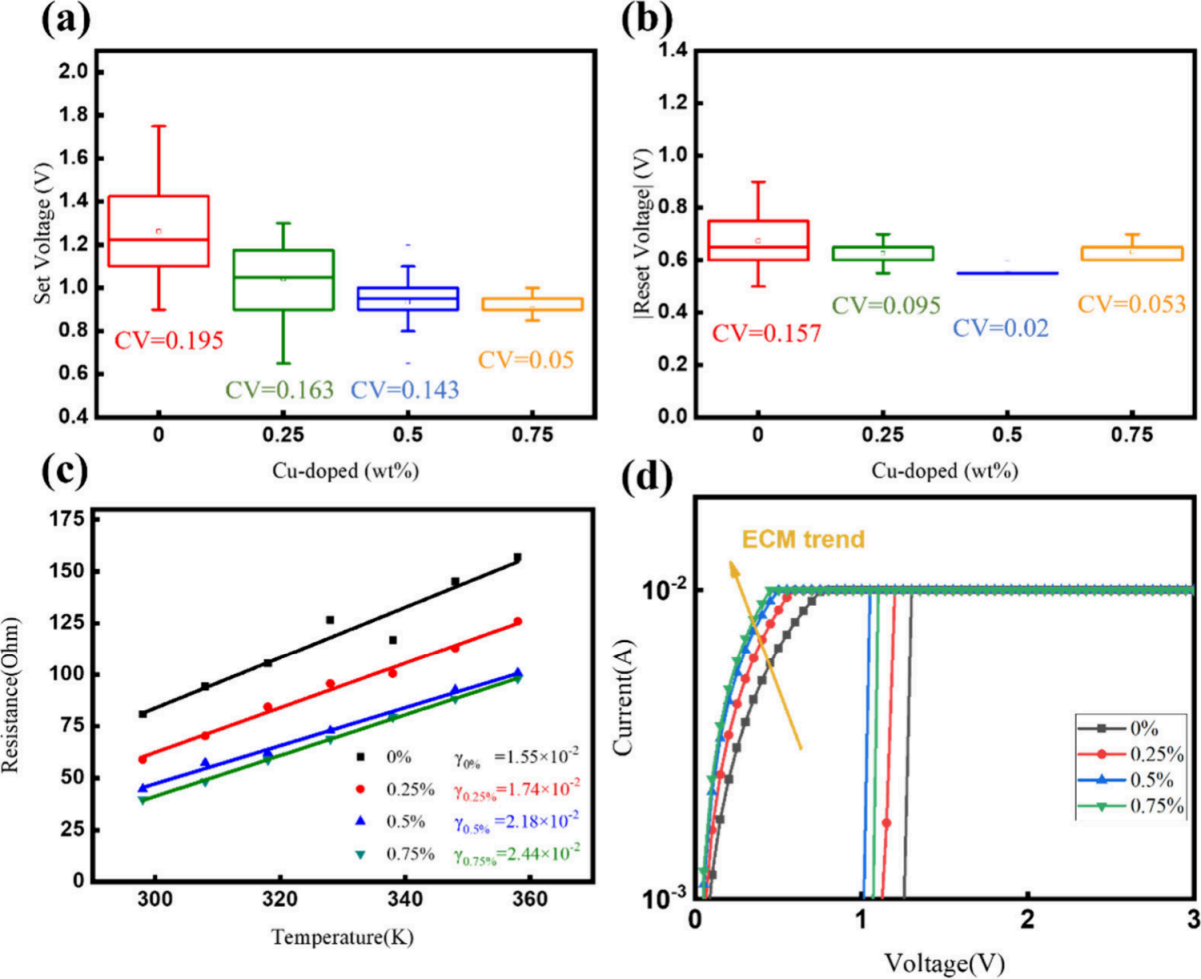
Stability, uniformity, and temperature-dependent analysis of CBRAM devices with copper-doped ZnO nanorods, confirming ECM-type operation. (a) Average set voltages and (b) reset voltages are represented as squares in the box plots, with coefficient of variation (CV) values also displayed. (c) Low-resistance state (LRS) resistance values for CBRAM devices with varying copper doping concentrations were measured at temperatures of 308, 318, 328, 338, 348, and 358 K, and plotted against temperature. (d) Observed trend is consistent with the characteristics of ECM-type behavior.

To assess operational consistency, we conducted a statistical analysis of the operation voltages over 100 cycles for the aforementioned CBRAM devices, with results listed in Table S1 of the Supporting Information. The average values and standard deviations are denoted as μ and σ, respectively.^44^ Panels a and b of Figure 4 illustrate the average operation voltages, represented as squares in the box plots. The coefficient of variation (CV), defined as σ /μ, serves as a standardized measure of data dispersion. We observed small CV values across all operation voltages, indicating good uniformity of our devices. Notably, the CV values of CBRAM devices with Cu-doped ZnO nanorods were lower than those of the corresponding CBRAM devices without Cu doping, indicating that copper ion doping facilitated the formation of a stable path for the conductive filaments in the switching layer, thereby reducing the operating voltage. This resulted in more uniform operating voltages, as the conductive filaments were able to form and rupture to an optimal extent. In particular, the CBRAM with 0.75% copper doping exhibited the most significant improvement. Further verification of this effect will be provided in subsequent studies, where the mechanism of copper ion doping in promoting uniform filament formation will be demonstrated and explained in Figure 6.

### 3.3 Mechanistic Analysis of Conductive Filament Formation

To further elucidate the nature of the conducting filament, we conducted a linear temperature-dependent R_LRS_ measurement, as eq 10.
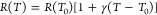
10In eq 10, *R*(*T*) represents the LRS resistance at temperature *T*, *R*(*T*_0_) the LRS resistance at room temperature T_0_, and γ signifies the temperature coefficient of resistance.^28,45−47^ Initially, the set process was carried out to switch the CBRAM device with a structure of Cu/TiW/Cu-doped ZnO nanorods/Pt to the LRS at room temperature. Subsequently, the device was heated, and *R*_LRS_ was measured at temperatures of 308, 318, 328, 338, 348, and 358 K. As shown in Figure 4c, the linear fitting curve reveals that *R*_LRS_ increases with increasing temperature. By comparing the temperature coefficients from the literature, listed in Table S2 of the Supporting Information, with our results, we found that the temperature coefficients for oxygen vacancy-based filaments were below 10^–2^ K^–1^, while those for Cu-based filaments were above 10^–2^ K^–1^. Through linear curve fitting, we obtained a temperature coefficient value of more than 1.55 × 10^–2^ K^–1^ for our device, which is consistent with the characteristics of Cu-based filaments. This suggests that copper atoms predominantly influence the filament composition, indicating that the electrochemical metallization (ECM) mechanism governs the switching behavior of our device. Furthermore, we observed that the value of γ increases with the copper doping concentration, indicating a stronger tendency toward the ECM conduction mechanism in the devices, resulting in lower resistance values of LRS as shown in Figure 4d. This tendency is associated with the number and distribution of oxygen vacancies. In comparison to the pure ZnO nanorods switching layer, the Cu-doped ZnO nanorods switching layers contain more oxygen vacancies, facilitating the diffusion of copper into the resistive switching layer.

**Figure 5:**
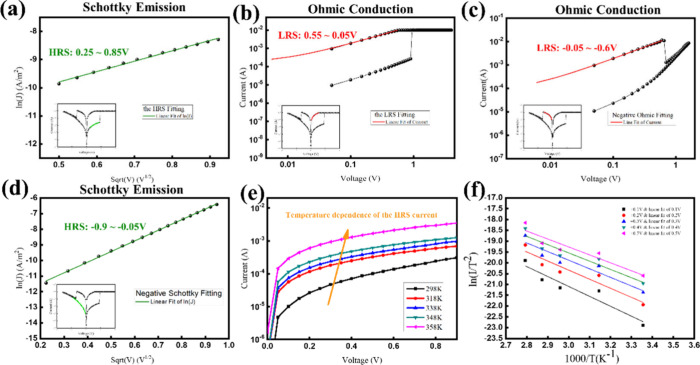
Fitted current curves illustrating the conduction mechanism of CBRAM with copper-doped ZnO nanorods. (a) High-resistance state (HRS) in the voltage range from 0.25 to 0.85 V. (b) Low-resistance state (LRS) in the range from 0.55 to 0.05 V. (c) LRS in the range from −0.05 to −0.6 V. (d) HRS in the range from −0.9 to −0.05 V. (e) Temperature dependence of the HRS current curve. (f) Linear fitting of current density as a function of temperature.

To further analyze the current conduction mechanism of the device, we performed fitting on the current characteristics curve of the 0.75% Cu-doped CBRAM and presented the results in Figure 5. The *I*–*V* characteristics of the CBRAM can be divided into four segments, primarily composed of Ohmic conduction and Schottky emission. Similar conduction mechanisms were observed in both positive and negative sweeps.^39^ In Figure 5a, the relationship between current density and applied voltage can be described by the Schottky emission equation, as shown in eq 11.
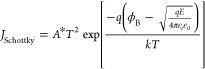
11To further elucidate the current conduction mechanism in the CBRAM device, we conducted a fitting analysis on the current characteristic curves of the 0.75% device, as illustrated in Figure 5. The *I*–*V* characteristics of the CBRAM device can be segmented into four distinct regions, predominantly governed by Ohmic conduction and Schottky emission. These conduction mechanisms are consistent in both positive and negative voltage sweeps. As depicted in Figure 5a, the relationship between current density and applied voltage adheres to the Schottky emission equation, exhibiting a strong fit. Furthermore, during positive voltage sweeps, the LRS conduction mechanism is primarily characterized by ohmic conduction, evidenced by the linear relationship between current density and applied voltage, as shown in Figure 5b. To substantiate the Schottky emission as the prevailing conduction mechanism, we investigated the temperature dependence of the HRS current. As illustrated in Figure 5e, the HRS current demonstrates a significant temperature dependence, increasing with rising temperature.^40^ According to the Schottky emission equation, a linear relationship is observed when the current density and temperature are rearranged in the specified form. This trend is corroborated by the data presented in Figure 5f, indicating that Schottky emission predominates the current conduction mechanism during the HRS DC voltage sweep.

**Figure 6:**
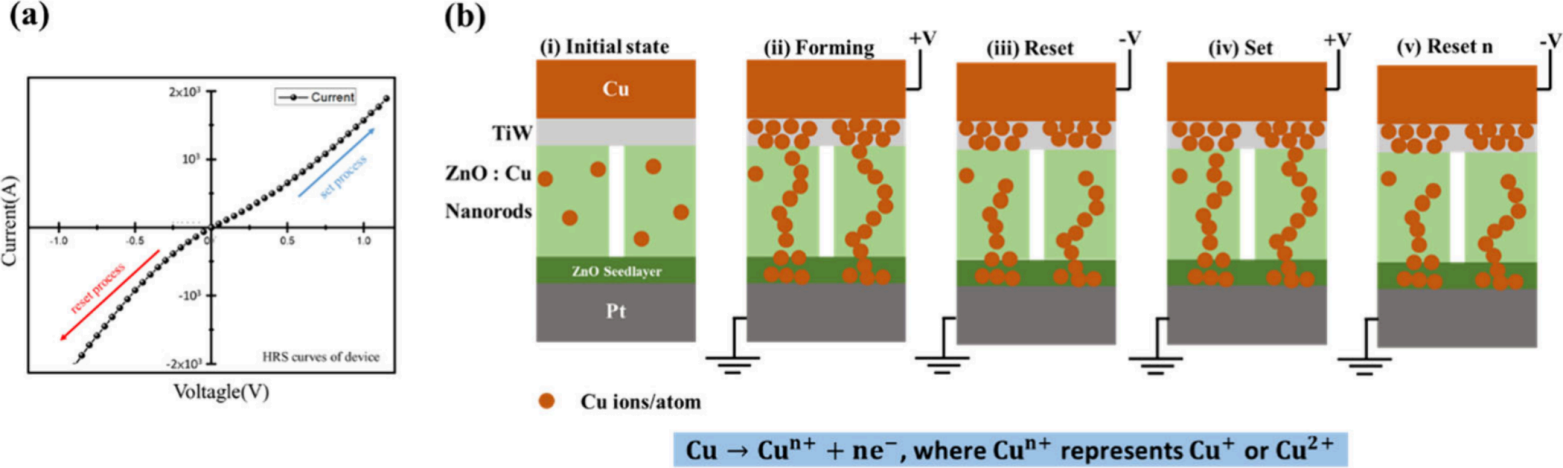
Operating principle of the CBRAM with copper-doped ZnO nanorods. (a) Symmetrical high-resistance state (HRS) curves observed during the reset and set processes. (b) Schematic illustration of the conduction mechanism, depicting the initial state, forming, reset, and set processes.

Integrating these findings, the resultant *I*–*V* curve is depicted in Figure 6a. Previous literature suggests that this *I*–*V* curve behavior may stem from the disruption of the conductive filament, either at the center of the switching layer or near the interface adjacent to the copper electrode.^41^ However, the study further indicates that the conductive filament tends to form in a conical shape, leading to fractures typically occurring at the anode or cathode interfaces rather than the central region. Integrating these insights, an operational schematic is presented in Figure 6b. In the initial state (i), oxygen vacancies and copper elements are present within the ZnO nanorods, facilitating the diffusion of copper ions. During the forming process, copper atoms from the upper electrode ionize into Cu^+^ ions and are injected into the switching layer. These Cu^+^ ions migrate toward the inert metal end under an applied electric field, gain electrons, and reduce back to copper atoms. This process results in the stacking of copper atoms along the nanorod boundaries, forming a metallic conductive filament, as illustrated in Figure 6b(ii), thereby transitioning the device into a LRS. During the negative voltage sweep, the copper atoms in the top conductive filament ionize into Cu^+^ ions^42,43^ and migrate back to the upper electrode, leading to the rupture of the filament and reverting the device to a HRS.^41^ The subsequent set and reset cycles involve continuous switching between high and low resistance states. The incorporation of the TiW layer effectively limits the influx of copper ions from the upper electrode into the switching layer, causing filament rupture and reformation to frequently occur along the same conductive filament pathway. During the reset process, the reverse bias applied to the copper electrode attracts Cu^+^ ions back to the copper electrode, resulting in filament rupture and the device switching back to HRS. Schottky emission is the predominant current conduction mechanism in this process, primarily associated with TiW/Cu:ZnO interface.^39^ In CBRAM devices without the inclusion of nanorod structures, the diffusion of copper ions is not physically constrained by the structure. This implies that the resulting conductive filaments formed by copper ions tend to be conical. Due to the randomness of copper ion diffusion, these conical filaments can vary significantly in size, being either relatively thick or thin. Thicker filaments require higher reset voltages, whereas thinner filaments require lower reset voltages. This variability leads to randomness in the reset voltage, and thicker filaments often face issues such as failure to reset or inability to reform after a high-voltage reset, thereby affecting the endurance of the device.

The incorporation of ZnO nanorod structures enhances the uniformity of conductive filament formation, resulting in reduced and stabilized forming and set/reset voltages. However, as shown in Figure 6b, this structure necessitates longer copper ion diffusion paths, complicating filament formation. Filaments are formed predominantly in regions with a higher copper ion concentration. Experimental findings indicate that copper ions doped within the nanorods act as intermediaries in the filament formation process. The optimal copper ion doping concentration minimizes the formation of ineffective filaments caused by diffusion limitations, thereby promoting efficient filament growth without necessitating excessively high ion concentrations. Furthermore, an increase in oxygen vacancy concentrations within the material facilitates copper ion diffusion, thereby reducing the bias voltage required to generate the electric field that drives filament formation. This mechanism effectively lowers both the forming and operating voltages.

The retention performance of CBRAM devices is shown in Figure S8 of the Supporting Information. Enhanced filament formation uniformity, attributed to copper ion doping, significantly improves resistance state retention in devices with copper doping concentrations of 0.5 and 0.75% during a 10 000 s test, compared to devices with lower doping levels. Additionally, uniformity testing, presented in Figure S9 of the Supporting Information, demonstrates consistent memory window distributions across four randomly sampled devices with 0.5 and 0.75% copper doping. A comparison with recent related studies is provided in Table 3. As shown in Table 3, our work demonstrates several advantages compared to previously reported studies on ZnO nanorod-based RRAM devices. First, the copper concentration in our process is significantly lower, reducing the risk of unintended contamination during fabrication and ensuring higher reliability. Furthermore, the set/reset voltage of our CBRAM is smaller than that reported in other studies, enabling more energy-efficient operation of the memory devices and associated circuitry. Our devices exhibit excellent performance in endurance and retention tests, as evidenced by great cycling stability and data retention compared to other studies summarized in Table 3. These results highlight the good reliability and promising potential of our approach for next-generation memory applications.

**Table 3:** Comparison of This Work to Other RRAM Devices Reported in the Literature^a^

RRAM device	*n*_Cu_ (%)	thickness of switching layer	*V*_set_ (V)	|*V*_rest_| (V)	on/off ratio (a.u.)	endurance (DC cycles)	retention (s)	RRAM type
Ag/ZnO:Cu/ITO^48^	2 mol %	none	1.8	0.02	∼10^6^	none	<10^3^	CBRAM
ITO/ZnO:Cu/Ag^48^	2 mol %	none	15	4	∼10^4^	none	>10^3^	OxRAM
Al/ZnO:Cu/Pt^22^	3 mol %	∼100 nm	2	0.5	∼5 × 10^1^	>400 cycles	10^4^	OxRAM
Ti/single CZO NRs/Ti^21^	4 atomic %	15 μm	>20	>20	∼10^3^	none	none	OxRAM
Al/ZnO:Cu/ITO^49^	5 mol %	50 nm	2.4	1.8	∼10^3^	none	10^6^	OxRAM
Au/ZnO:Cu/FTO^50^	9 mol %	100 nm	2	4	>10^1^	none	none	OxRAM
Cu/TiW/CZO NRs/Pt (this work)	<1 mol %	∼450 nm	0.9	0.6	∼5 × 10^1^	>3000 cycles	>10^4^	CBRAM

a The switching layers in these studies also utilize copper-doped zinc oxide materials, primarily in the form of thin-film structures. The table demonstrates that our CBRAM devices with Cu-doped ZnO nanorods achieve lower operating voltages and higher DC sweep endurance, along with satisfactory retention, attributed to the nanorod structure and low copper doping concentration. However, the enhancement of the memory window in our devices is less pronounced compared to others, which may be attributed to the low copper doping concentration, limited increase in thin-film resistivity, or the formation of new phases such as CuO at higher copper doping levels.

## 4 Conclusion

In conclusion, this study successfully employed a hydrothermal method with copper ion doping to fabricate high-quality copper-doped ZnO nanorods as the switching layer in CBRAM devices, achieving significant improvements in die-to-die uniformity and memory stability. The integration of ZnO thin films with nanorod structures effectively modulates the morphology of conductive filaments in the switching layer. Specifically, the nanorods constrain the filament thickness within their physical width, preventing the formation of excessively thick filaments. Filament rupture during HRS/LRS switching predominantly occurs at the TiW/ZnO nanorod interface, where the nanorod structure ensures consistent filament disconnection and reconnection, further enhancing operational stability. These structural and functional enhancements are demonstrated by reduced set/reset voltages, improved energy efficiency, and enhanced operational stability. Copper ion doping plays a critical role in increasing the oxygen vacancy concentration within the nanorods, thereby facilitating copper ion diffusion. The uniformly doped copper ions participate in filament formation, reducing dependence upon electrode-derived ions. By acting as intermediaries, these doped ions enable easier filament growth while mitigating excessive thinning and brittleness, thus improving filament uniformity. In comparison to undoped ZnO nanorods, the incorporation of 0.75% copper doping significantly extended the operational cycles of the memory devices from 120 to 3100 and increased retention time from 3000 to over 10 000 s. Memory stability, as measured by the coefficient of variation, improved by over 70% for the set voltage and approximately 60% for the reset voltage, while the memory window expanded by around 60%. The effects of copper ion doping on ZnO crystallinity and oxygen bonding were systematically analyzed, highlighting their contribution to enhanced operational uniformity and reduced operating voltages in CBRAM devices. These advancements are attributed to the synergistic combination of ZnO nanorod application and copper ion doping, as neither approach independently achieves such substantial improvements. Furthermore, the hydrothermal fabrication process, conducted at temperatures below 100 °C, represents a notable innovation. This low-temperature method not only minimizes energy consumption but also expands compatibility with flexible substrates and advanced integration technologies. The simplicity, scalability, and low-temperature nature of this fabrication process make it a promising approach for significantly enhancing CBRAM performance, positioning it as a viable candidate for high-density CIM storage applications.

## Supporting Information Available

Top-view and cross-sectional SEM images of ZnO nanorods grown under different growth parameters, XRD patterns of ZnO nanorods grown at various copper doping concentrations, endurance test results of CBRAM devices, XPS analysis results of ZnO nanorods, standard deviation of set/rest voltages for CBRAM devices with different copper doping concentrations, comparison of the temperature coefficients obtained in this study to those reported in other literature, retention test results for CBRAM devices, memory window box plot of the devices, and CV values of the memory window for the devices (PDF)
