# Supplementary material for: Enhancing Stability and Performance of Conductive Bridge Random Access Memory: Use of a Copper-Doped ZnO Nanorod-Embedded Switching Layer
Source: ACS Appl Mater Interfaces. 2025 Feb 12;17(8):12495–506. doi: 10.1021/acsami.4c16223 (PMC11874029; doi:10.1021/acsami.4c16223)
Supplement: Supplementary file 1 — am4c16223_si_001.pdf [file am4c16223_si_001.pdf]

# Supporting Information

# Enhancing Stability and Performance of Conductive Bridge Random Access Memory: Use of Copper-Doped ZnO Nanorod-Embedded Switching Layer

*Po-Tsun Liu[1*], Yu-Chuan Chiu[1], Chih-Chieh Hsu[2], Kai-Jhih Gan[2], Dun-Bao Ruan[2],*

*Sheng-Jie Su[1], Shu-Wei Chang[1]*

[1]Department of Photonics and Institute of Electro-Optical Engineering, National Yang Ming Chiao Tung University, Hsinchu 300093, Taiwan

[2] Institute of Electronics, National Yang Ming Chiao Tung University, Hsinchu 300093, Taiwan

*Address correspondence to: ptliu@nycu.edu.tw

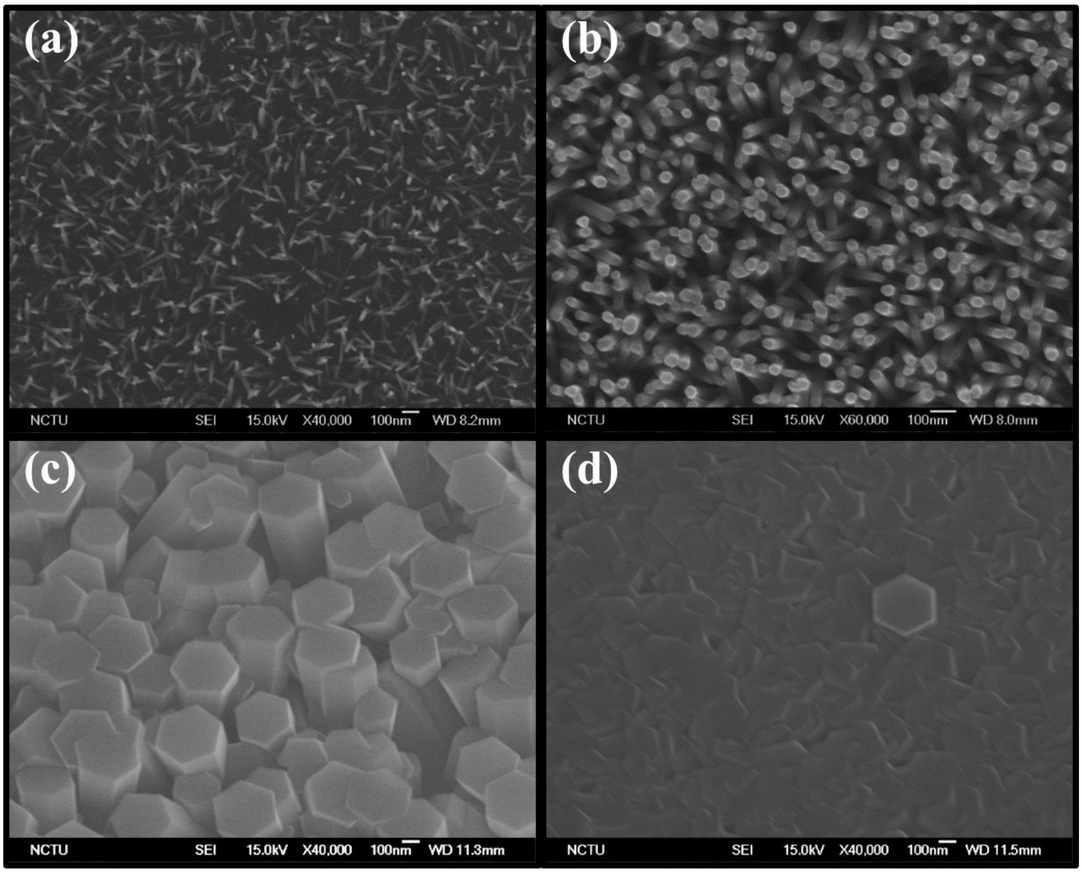


**Figure S1.** FESEM of ZnO nanorods grown with different concentrations of zinc acetate molarity. (a) 0.005M (b) 0.02M (c) 0.05M (d) 0.08M.

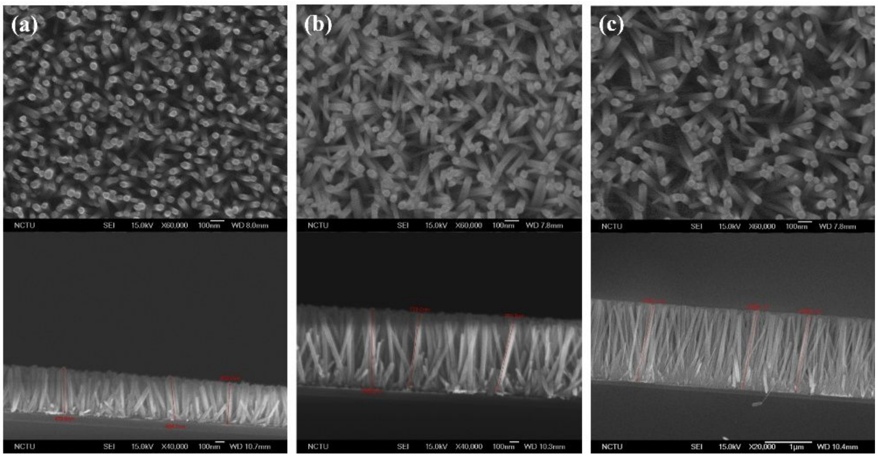


**Figure S2. FESEM cross-sectional and top-view images of ZnO nanorods grown at a zinc acetate concentration of 0.02 M for different durations.** (a) 30 minutes, yielding nanorods with an approximate length of 450 nm. (b) 60 minutes, yielding nanorods with an approximate length of 790 nm. (c) 90 minutes, yielding nanorods with an approximate length of 1600 nm.

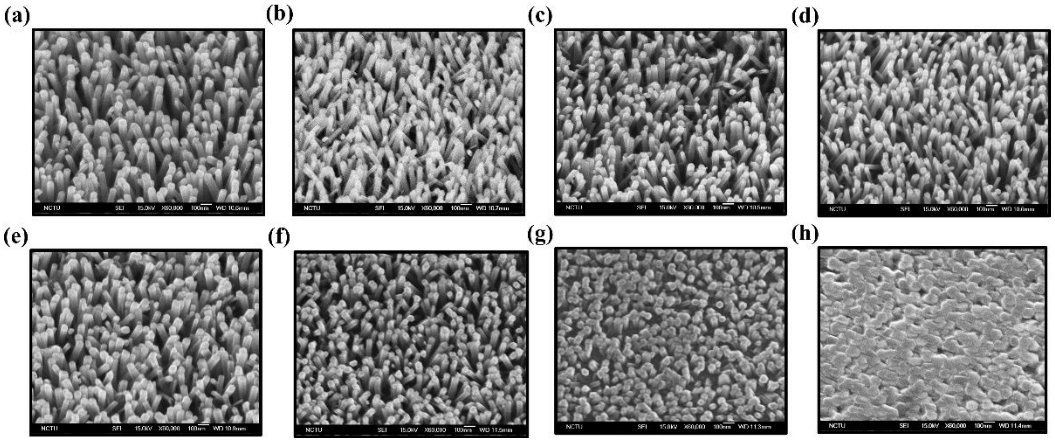

**Figure S3. FESEM images of ZnO nanorods doped with varying copper concentrations, captured at a 45° tilt.** (a) 0%, (b) 0.1%, (c) 0.25%, (d) 0.5%, (e) 0.75%, (f) 1%, (g) 1.5%, and (h) 2%.

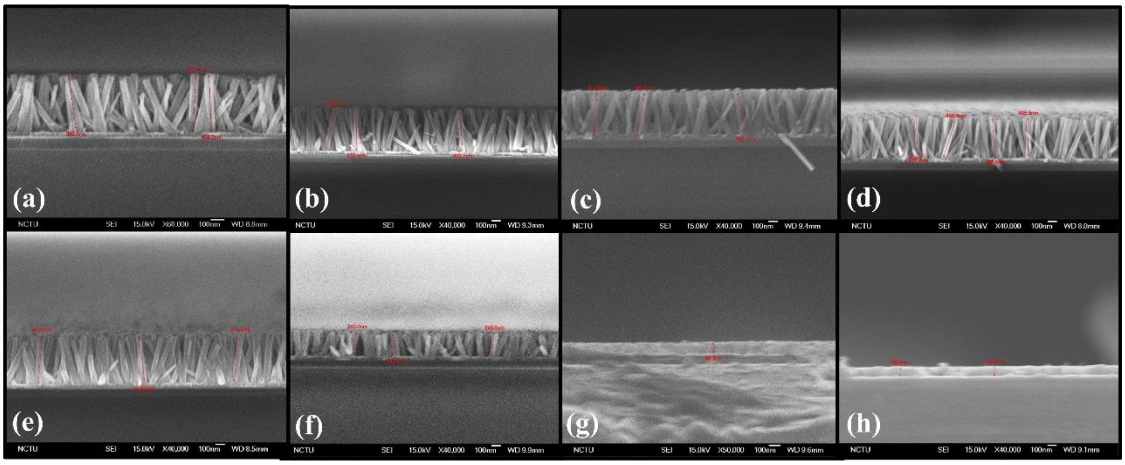


**Figure S4. FESEM cross-sectional images and corresponding length measurements of ZnO nanorods doped with varying copper concentrations.** The nanorods were synthesized using a zinc acetate concentration of 0.02 M and a water bath method for 30 minutes. (a) 0%, 420 nm; (b) 0.1%, 430 nm; (c) 0.25%, 460 nm; (d) 0.5%, 420 nm; (e) 0.75%, 500 nm; (f) 1%, 240 nm; (g) 1.5%, 90 nm; (h) 2%, 100 nm.

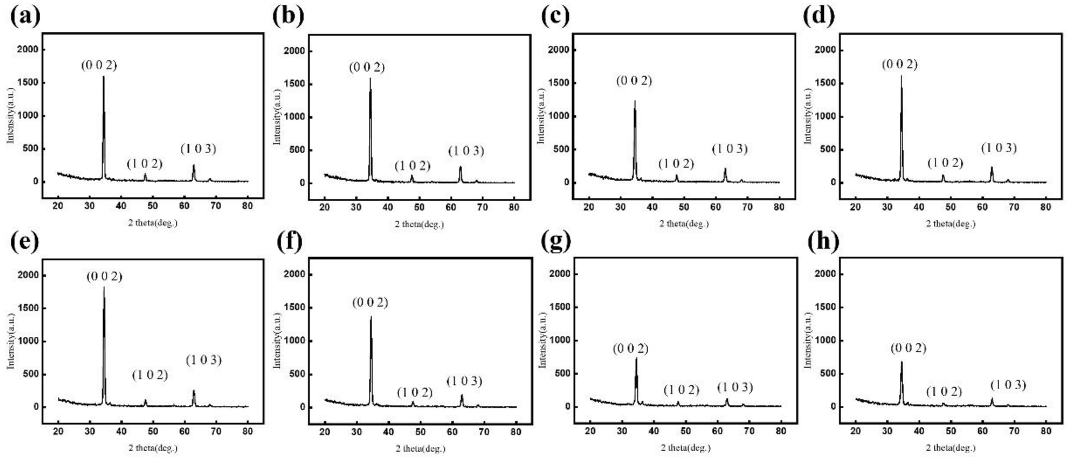


Figure S5. **X-ray diffraction patterns of ZnO nanorods doped with varying copper concentrations.** (a) 0%. (b) 0.1%. (c) 0.25%. (d) 0.5%. (e) 0.75%. (f) 1%. (g) 1.5%. (h) 2%. The nanorods were synthesized using a zinc acetate concentration of 0.02 M and a water bath method for 30 minutes.

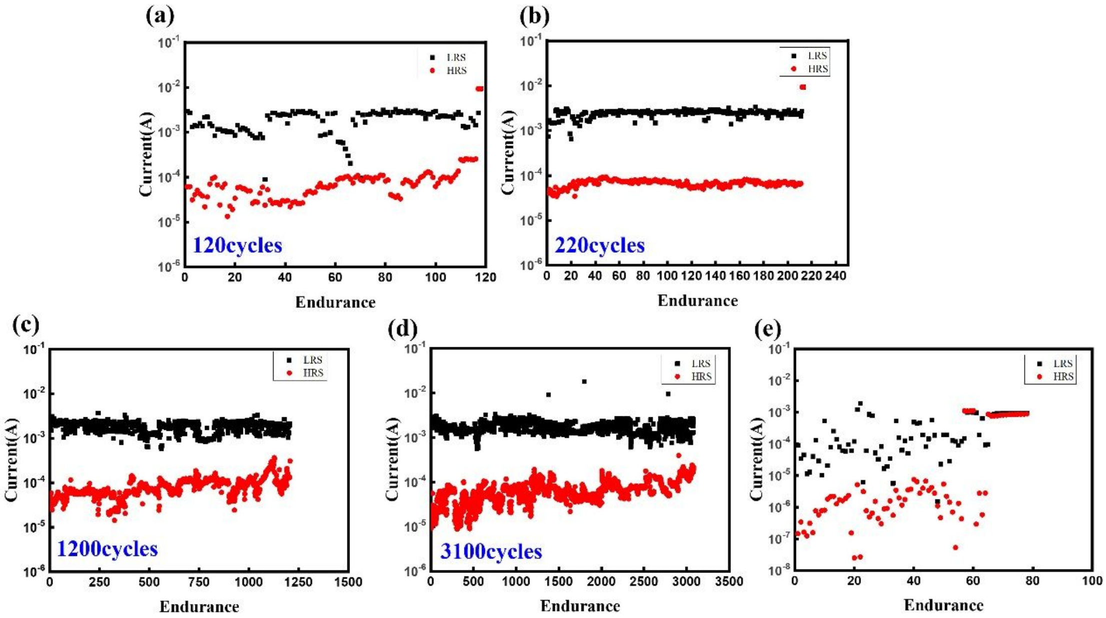


**Figure S6.** Current levels of the high-resistance state (HRS) and low-resistance state (LRS) for Cu-doped ZnO nanorods-based CBRAM devices with varying copper doping concentrations during the cycling endurance test. The reading voltage was set to -0.1 V. (a) 0%, (b) 0.25%, (c) 0.5%, (d) 0.75%, and (e) 1%.

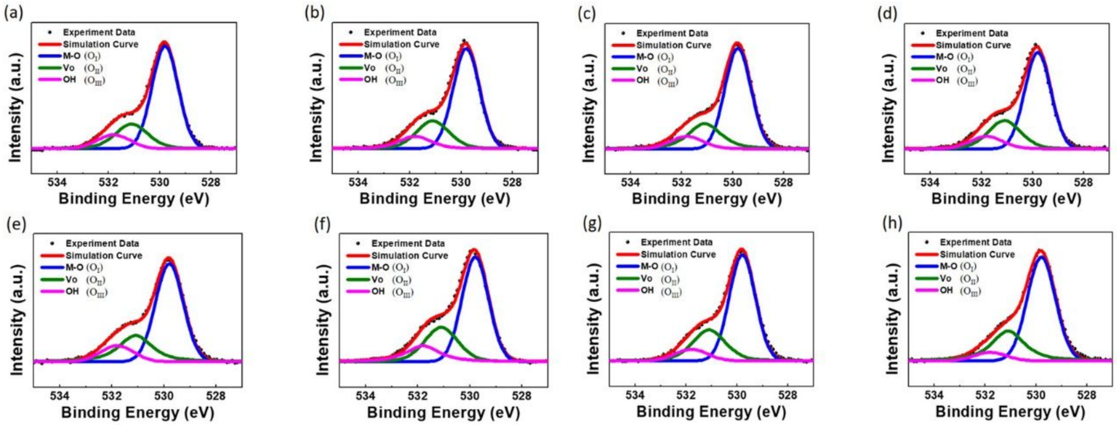


**Figure S7**. XPS analysis of the $O_{1s}$ electronic orbital bonding in ZnO nanorods with varying copper doping concentrations. (a) Pure ZnO, (b) 0.1%, (c) 0.25%, (d) 0.5%, (e) 0.75%, (f) 1%, (g) 1.5%, and (h) 2%.

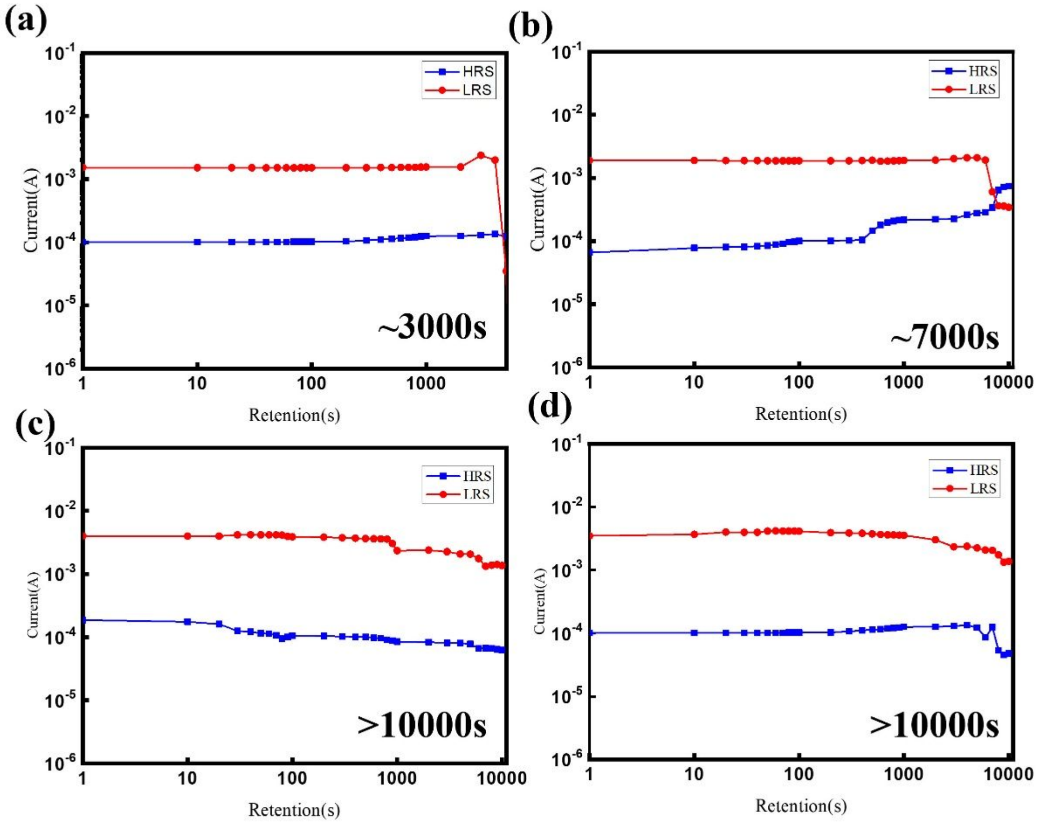


**Figure S8.** Retention tests of Cu-doped ZnO nanorod-based CBRAM devices with varying copper doping concentrations. The HRS and LRS currents were recorded over time to evaluate retention performance. (a) Pure ZnO, (b) 0.25%, (c) 0.5%, and (d) 0.75%.

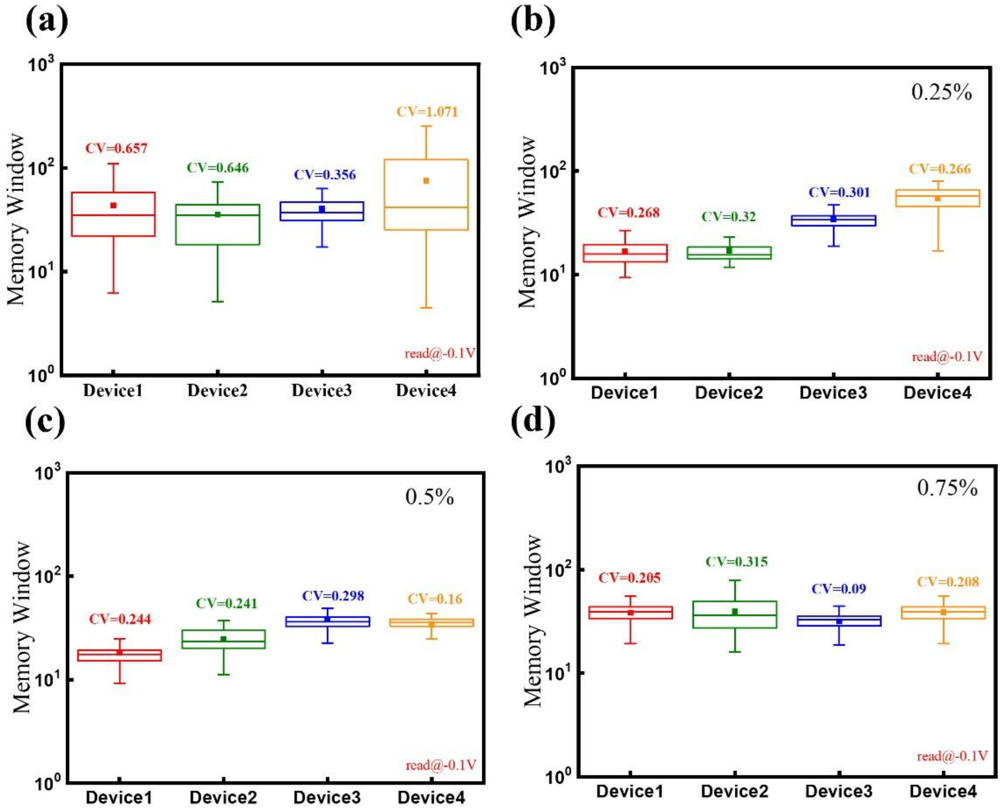


**Figure S9.** Box plots of the memory window for Cu-doped ZnO nanorod-based CBRAM devices with varying copper doping concentrations, measured over 100 operation cycles. The coefficient of variation (CV) values corresponding to these results are presented in Table S3. (a) Pure ZnO, (b) 0.25%, (c) 0.5%, and (d) 0.75%.

| **Percentages of Cu-doped ZnO nanorods (%)** | $V_{set}$ | $\|V_{reset}\|$ |
|---|---|---|
| 0% | σ=0.246V<br>μ=1.262V<br>CV=0.195 | σ=0.106V<br>μ=0.673V<br>CV=0.157 |
| 0.25% | σ=0.170V<br>μ=1.043V<br>CV=0.163 | σ=0.060V<br>μ=0.628V<br>CV=0.095 |
| 0.5% | σ=0.134V<br>μ=0.935V<br>CV=0.143 | σ=0.011V<br>μ=0.553V<br>CV=0.020 |
| 0.75% | σ=0.046V<br>μ=0.905V<br>CV=0.050 | σ=0.034V<br>μ=0.633V<br>CV=0.053 |

**Table S1.** Set and reset voltages of Cu-doped ZnO nanorods-based CBRAM devices with varying copper doping concentrations, calculated over 100 operation cycles. Here, σ represents the standard deviation, μ denotes the mean value, and CV refers to the coefficient of variation.

| **Device Structure** | **γ(K$^{-1}$)** | **RRAM type** |
|---|---|---|
| Cu/$ZrO_2$/TiN [24] | $1.8 \times 10^{-2}$ | CBRAM |
| Pt/Ti/$Ta_2O_5$/Pt [42] | $5.7 \times 10^{-3}$ | OxRAM |
| Pt/$NiFe_2O_4$/Pt [36] | $5.6 \times 10^{-3}$ | OxRAM |
| TiN/$TiO_2$/bulk-Cu [37] | $1.1 \times 10^{-2}$ | CBRAM |
| Cu/TiW/ZnO/Pt [this work] | $> 1.5 \times 10^{-3}$ | CBRAM |

**Table S2.** Comparison of the temperature coefficients obtained in this study with those reported in previous literatures.

| | Device1 | Device2 | Device3 | Device4 | Average Value |
|---|---|---|---|---|---|
| **0%** | 0.657 | 0.646 | 0.357 | 1.071 | 0.683 |
| **0.25%** | 0.268 | 0.320 | 0.301 | 0.266 | 0.289 |
| **0.5%** | 0.244 | 0.241 | 0.298 | 0.160 | 0.236 |
| **0.75%** | 0.205 | 0.315 | 0.209 | 0.208 | 0.234 |

**Table S3.** Coefficient of variation (CV) values for the memory window of the four Cu-doped ZnO nanorods-based CBRAM devices with varying copper doping concentrations, as presented in **Figure S9**.
